# Non-Contact Heart Rate and Blood Pressure Estimations from Video Analysis and Machine Learning Modelling Applied to Food Sensory Responses: A Case Study for Chocolate

**DOI:** 10.3390/s18061802

**Published:** 2018-06-03

**Authors:** Claudia Gonzalez Viejo, Sigfredo Fuentes, Damir D. Torrico, Frank R. Dunshea

**Affiliations:** School of Agriculture and Food, Faculty of Veterinary and Agricultural Sciences, University of Melbourne, Parkville, VIC 3010, Australia; cgonzalez2@unimelb.edu.au (C.G.V.); damir.torrico@unimelb.edu.au (D.D.T.); fdunshea@unimelb.edu.au (F.R.D.)

**Keywords:** blood pressure, computer vision, heart rate, machine learning modelling, non-intrusive sensors

## Abstract

Traditional methods to assess heart rate (HR) and blood pressure (BP) are intrusive and can affect results in sensory analysis of food as participants are aware of the sensors. This paper aims to validate a non-contact method to measure HR using the photoplethysmography (PPG) technique and to develop models to predict the real HR and BP based on raw video analysis (RVA) with an example application in chocolate consumption using machine learning (ML). The RVA used a computer vision algorithm based on luminosity changes on the different RGB color channels using three face-regions (forehead and both cheeks). To validate the proposed method and ML models, a home oscillometric monitor and a finger sensor were used. Results showed high correlations with the G color channel (R^2^ = 0.83). Two ML models were developed using three face-regions: (i) Model 1 to predict HR and BP using the RVA outputs with R = 0.85 and (ii) Model 2 based on time-series prediction with HR, magnitude and luminosity from RVA inputs to HR values every second with R = 0.97. An application for the sensory analysis of chocolate showed significant correlations between changes in HR and BP with chocolate hardness and purchase intention.

## 1. Introduction

Heart rate (HR) is defined as the number of heart beats within a certain interval of time, usually measured as beats per minute (BPM) [1]. Besides medical applications, this is one of the most common biometrics used for user authentication [2] and, more recently, to assess physiological responses of consumers to different stimuli, which can be associated to responses from the autonomic nervous system (ANS) to consumption and visual assessment of food and beverages [3,4,5,6]. However, these biometrics usually involve intrusive techniques such as the electrocardiogram (ECG), and oscillometry, which require sensors to be attached to subjects. There are other methods such as the photoplethysmography (PPG), which uses a pulse oximeter and is considered by some authors as non-invasive. However, this sensor still requires to be in contact with the skin of the subject. Some non-intrusive methods have been developed based on the PPG principle as a validation method to develop computer vision analysis to obtain HR from videos [7,8,9].

The electrocardiogram (ECG) method is the most commonly used for medical purposes and it requires the attachment of one or more sensors to either the chest or the four extremities of subjects, this method measures the electrical activity of the heart that occurs during the cardiac cycle. This activity is registered and printed as a signal composed of P-waves that represent the depolarization of the atriums, PR interval that represents the delay of the impulse in the atrioventricular node, the QRS complex that consists of the electrical current generated during the depolarization and ventricular contraction, and the ST-segment and T-waves that represent the ventricular repolarization [8,10]. Oscillometry is the method usually used for ambulatory or at home monitoring which requires to be attached to the arm or wrist and is able to measure blood pressure and HR by inflating the cuff until it reaches the occlusion of blood flow in the artery, once this is achieved, it is released, and the device is able to measure the oscillations in the arterial wall produced when the blood starts flowing. This allows to obtain the measurements for blood pressure (systolic and diastolic) as well as the HR by measuring the time between the oscillometric pulses [11,12].

The PPG, method consists of a device called pulse oximeter, which is attached to either the finger or earlobe, which measures the oxygen saturation in the blood. The device emits both red and infrared lights, and measures the amount of light absorbed or reflected by the blood in the earlobe or finger [13,14]. This method can also use green light, which, unlike the red and infrared lights, is absorbed by the oxy- and deoxyhemoglobin and, therefore, can only measure the blood flow in the skin surface. Some authors have developed methods based on PPG to calculate HR using videos from the face of the subjects. For example, Takano and Ohta [7] developed a technique using time-lapse images and based on the measurement of the brightness and Jain et al. [15] developed a method to estimate HR and blood pressure using videos and principal component analysis to extract the PPG signal. Carvalho et al. [16] calculated HR using a webcam to record videos preprocessed using the Eulerian Magnification algorithm (EMA) developed by the Massachusetts Institute of Technology (MIT) [17] and an algorithm written in Matlab^®^ (R2013a, Mathworks Inc., Matick, MA, USA) to extract the signal from RBG components from videos. Jensen and Hannemose [18] used a similar method, but without the EMA pre-processing. Al-Naji et al. [19,20] used an improved version of the EMA to enhance the HR signal, but based on motion of areas such as chest and wrist. Wei et al. [21] also used a webcam and analyzed videos with a technique based on Laplacian Eigenmap and RBG channels. Lewandowska et al. [22] proposed a method using a webcam and analyzing the signal combining the red and green channels and using independent component analysis and principal component analysis obtaining similar results with both methods. On the other hand, a commercial software, FaceReader™ (ver. 7.0, Noldus Information Technology, Wageningen, The Netherlands), that is able to measure HR using videos is available and it is based on the same principle of color changes that is then related to HR [23].

The importance of the development of non-intrusive methods relies on the influence and bias that the contact sensors have on the physiological responses of the subject as they may influence their HR [5] and blood pressure (BP) responses due to awareness of having sensors attached to their bodies. This effect of the influence of the awareness of wearing sensors and/or being monitored was studied by Frelih et al. [24], who found that certainly the use of sensors attached to the body had a significant effect on the participants experience. The application of these techniques is of great importance to assess HR as part of the responses from the ANS to different stimuli such as food, beverages, packaging and other stimuli such as imagery and videos, which combined with other ANS responses [3,25], may be associated to consumers acceptability, preference and quality perception of different food products such as beer and chocolate for example [4,5]. Apart from the applicability to obtain more information about the unconscious responses from consumers, this along with machine learning algorithms, aids in the development of more objective and efficient sensory methods. The implementation of non-intrusive techniques for ANS responses allows to have less time-consuming and more cost-effective techniques to predict different conscious responses. Some researchers have already associated HR changes and emotional responses using different stimuli such as images, real-life situations, instructing to do facial action tasks and using film clips, obtaining as results a positive relationship between HR and anger, anxiety, embarrassment, fear, sadness and happiness, and a negative relationship between HR and disgust, affection and pleasure [3,26,27,28,29]. With respect to liking, an increase in HR has been observed when children taste food products that they dislike and decrease in HR when they like the smell of a food, furthermore, a decrease in HR was observed when young adults assess products that they like visually [25]. However, there are no previous studies including BP as a physiological response to associate with the aforementioned responses.

This paper aims to develop and validate a non-intrusive or non-contact method to measure HR using the PPG technique, which has been validated using a home oscillometric HR apparatus to obtain targeted continuous HR data from participants. The proposed method was developed using machine learning algorithms to predict the real HR and BP as targets using regression algorithms and analysis of videos from participants as inputs. The models will be targeted to predict the raw signal of the luminosity changes and calculated HR every second using a time-series machine learning algorithm (TSML). Furthermore, the non-intrusive method developed to measure HR from the raw video analysis (RVA) was compared to two intrusive methods used for validation and two non-intrusive methods based on video analysis: (i) an OMRON HEM-790IT oscillometric blood pressure and HR monitor (OMRON Corporation, Kyoto, Japan), (ii) an open-source pulse sensor (PPG, World Famous Electronics llc, New York, NY, USA) controlled by an Arduino^®^ board (Arduino, Ivrea, Italy) and attached to the finger, (iii) HR outputs from FaceReader™ software, and (iv) the magnified video analysis (MVA) pre-processing the videos using the Eulerian Magnification Algorithm (EMA), respectively. Based on the results obtained from this study, the finger sensor had a high correlation with the oscillometric monitor, while FaceReader™ did not correlate at all with the latter. The video analysis algorithm had a high correlation when applying a correction factor when using the green channel in the RGB components, and when analyzing the forehead and both cheeks. Furthermore, it was possible to develop highly accurate machine learning models using artificial neural networks (ANN) and the non-linear autoregressive with exogenous input (NARX) algorithm to predict the raw signal of the luminosity changes and HR in every second of the videos as well as a highly accurate model using regression algorithms to predict the average of HR and blood pressure of each person per video. Finally, a case study of the application of the proposed non-intrusive method is presented using chocolate samples in a consumer test showing the correlation between the physiological (HR and BP) and self-reported responses.

## 2. Materials and Methods

### 2.1. Data Gathering Session Setup

**Figure 1:**
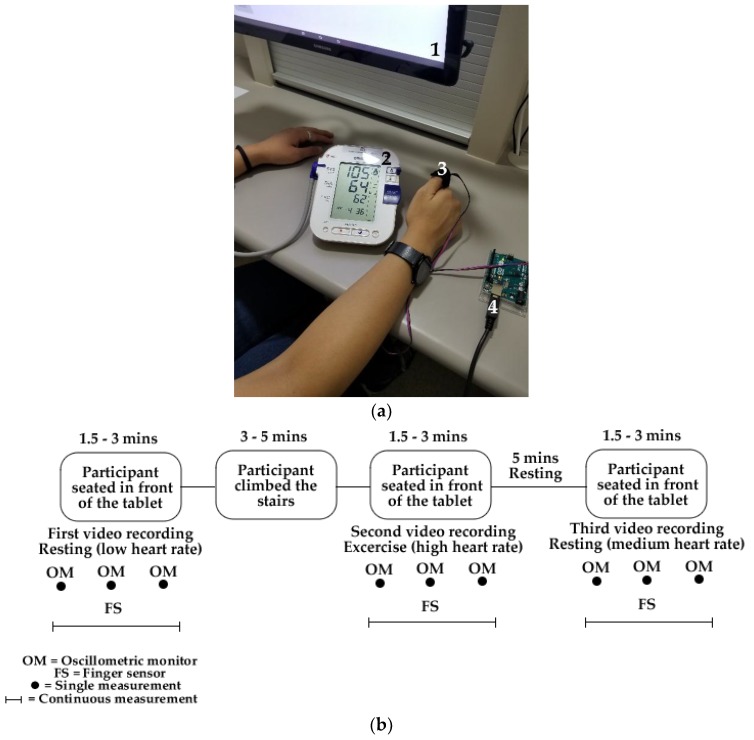
Data gathering set-up showing: (**a**) data acquisition system displaying: (1) the tablet with the Bio-sensory application for video acquisition, (2) oscillometric HR and blood pressure monitor and (3) finger sensor controlled by (4) an Arduino^®^ board, and (**b**) diagram of the protocol for the data and video acquisition.

A session with 15 healthy adult participants between 20 and 38 years old was conducted using an integrated camera system controlled by a bio-sensory application (App) designed for Android tablets (Google; Open Handset Alliance, Mountain View, CA, USA) developed by the sensory group from the School of Agriculture and Food, Faculty of Veterinary and Agricultural Sciences, The University of Melbourne. The integrated camera (1920 × 1080 pixels and 15 frames per second) along with the App are able to record RGB videos form the participants while conducting a sensory test in sensory booths [5]. The session was conducted in the sensory laboratory located at The University of Melbourne, Australia (Parkville campus) with controlled temperature (24–26 °C). Participants were asked to sit in individual sensory booths with uniform white lighting conditions and within 30 to 45 cm from the tablet using the App and were instructed to face at a blank screen in the tablet PCs. In the meantime, they were wearing a Food and Drug Administration (FDA)-approved oscillometric BP and HR monitor OMRON HEM-790IT that was activated manually to obtain a measure at the start, middle and end of the video acquisition. This oscillometric monitor has an accuracy for BP of ±3 mmHg and ±5% for HR according to the specifications of the device. In parallel, a finger pulse sensor controlled by Arduino^®^ (Arduino, Ivrea, Italy) was attached to the thumb of the participants to obtain HR continuously throughout the duration of the video (Figure 1a). The placement of the sensor in the thumb was determined after a previous test in which the most accurate results compared with the oscillometric monitor were obtained (data not shown), some references also mention the use of pulse oximeters on the thumb [30,31]. After getting the initial resting videos and HR-BP data from all participants, they were asked to climb the building stairs from the ground up to the fourth floor (3–5 min). After this activity, they returned to the booths and repeated the initial instructions to obtain videos and real HR data after physical activity. Finally, a third video was taken after 5 min resting to get the values from medium HR levels. Therefore, a total of three videos of 1.5 to 3 min each per person (*n* = 45) were obtained to validate the proposed method across a wide range of HR. Figure 1b shows a diagram of the described protocol for a better understanding.

### 2.2. Data Analysis and Computer Vision Algorithms

**Figure 2:**
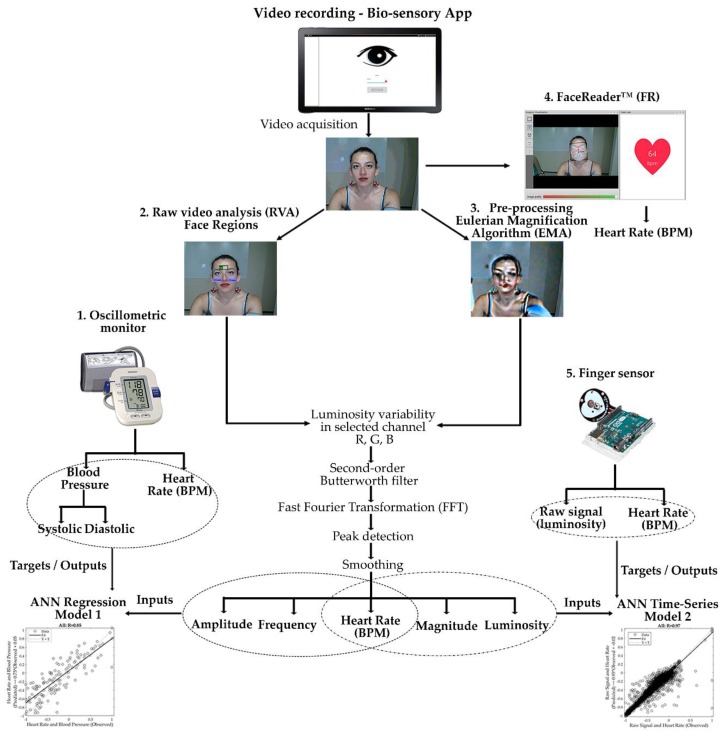
Flow diagram showing the data acquisition, analysis and outputs of the five methods presented to measure HR: (1) oscillometric monitor, (2) raw video analysis (RVA), (3) pre-processing using the Eulerian Magnification Algorithm (EMA), followed by the magnified video analysis (MVA) (4) FaceReader™ software, and (5) finger sensor. The diagram also depicts the inputs and targets/outputs used to develop the machine learning models. Abbreviations: ANN = Artificial Neural Networks, BPM = beats per minute.

All videos were analyzed using three techniques: (i) FaceReader™ 7.0 software (Noldus Information Technology, Wageningen, The Netherlands) (FR), (ii) pre-processing of videos by magnifying the color and luminosity changes using the Eulerian Magnification Algorithm (EMA) proposed by the Massachusetts Institute of Technology (MIT, Boston, MA, USA) [17], followed by the HR calculation using the proposed algorithm (Magnified Video Analysis: MVA), and (iii) using the raw videos (RVA) obtained from the tablets. Videos from techniques (ii) and (iii) were then analyzed using a customized computer vision algorithm written in Matlab^®^ ver. R2018a (Mathworks Inc., Matick, MA, USA). This algorithm works automatically and manually with the manual cropping of videos being used initially to analyze three different face regions: (i) forehead, (ii) right cheek and (iii) left cheek to find if any had a higher accuracy or if the combination of the three gave a better response. The cropped areas were rectangles within the range of 120–150 × 50–70 pixels for the forehead and 50–80 × 60–90 pixels for the cheeks. This process can be automated by adapting the point tracker algorithm based on the Kanade-Lucas-Tomasi (KLT) algorithm in Matlab^®^ used for feature tracking along with the Ground Truth Labeler found in the Matlab® Automated Driving System Toolbox 1.2 (Mathworks Inc., Matick, MA, USA) [32]. However, for the research reported here, the areas were cropped manually to assess the accuracy of the codes and avoid bias that might be related to the automatic process. The customized algorithm used to assess HR for both the RVA and the MVA, is able to measure luminosity changes in any of the RBG channels, therefore, the three channels were tested to assess the most accurate option. Once the signal was acquired, the algorithm applies a second-order Butterworth filter, the cutoff frequencies were set to 50 and 190 BPM (0.83 and 3.17 Hz) and a fast Fourier transformation (FFT) is applied for the transformation of the time signal to frequency. The settings were set to three seconds of stabilization time and one cut start second. The outputs obtained from this algorithm are the luminosity changes, HR values in BPM every 0.5 s, magnitude using the FFT, and the amplitude and frequency of each peak detected in the raw signal of the luminosity changes using the ‘findpeaks’ customized function [33]. The values are calculated from the maximum peak detected in the selected face region. Figure 2 shows the diagram with all the methods used in this study for more clarity of the analysis and modelling process.

The performance based on the elapsed time (s), Central Processing Unit (CPU) maximum (Max) and minimum (Min) temperatures, memory used (MB) and CPU usage (%) from the personal computers (PC) used were obtained by Matlab^®^ specific functions. Three different PCs from two brands (HP and Alienware) with distinct number of cores (two, four and ten) and memory (8 GB and 32 GB) were used. The performances were compared also with and without the engagement of the parallel pool option in Matlab^®^. The CPU temperatures were measured using the RealTemp 3.7 monitoring application (TechPowerUp, Cochrane, AB, Canada) designed for Intel processors, all other performance results were obtained using different built-in Matlab^®^ functions. For easier comparative purposes, the data from each performance category was normalized and colored to be presented on matrix graphic forms for easier comparison, this can be found as Supplementary Material (Figure S1).

### 2.3. Statistical Analysis and Machine Learning Modelling

Data from the oscillometric monitor, finger sensor and from the processed videos using different settings and the two methods (RVA and MVA) were analyzed statistically using Pearson linear correlation method (y = ax + b) [34,35] using the Matlab Curve Fitting Toolbox™ 3.5.6 (Mathworks Inc., Matick, MA, USA). Values of determination coefficients (R^2^), root mean squared error (RMSE), and statistical significance (criteria: *p* < 0.05) were used for fitting assessments. The *p*-value was calculated using the CoStat ver. 6.45 (CoHort Software, Monterey, CA, USA) software. For results obtained using the videos analyzed with Matlab^®^, a correction factor was applied as follows: low blood pressure < 75 BPM − 15 BPM, medium range 76–90 BPM with no correction applied and high range >91 BPM + 15 BPM. To determine the correction factor of ±15, the average of the difference between the lowest and highest HR values (delta) of all videos was calculated. This correction factor may be easily added to the RVA Matlab^®^ algorithm to accelerate and simplify the process instead of pre-processing the videos using different magnification settings according to the real HR as in the EMA. However, this correction factor is presented as an option to use the RVA when the real values of HR of the subject are known. Therefore, as further described, machine learning models were developed to predict the real HR and BP values to calculate these responses without the need to know the real values (measured with a contact device) of the subjects.

**Figure 3:**
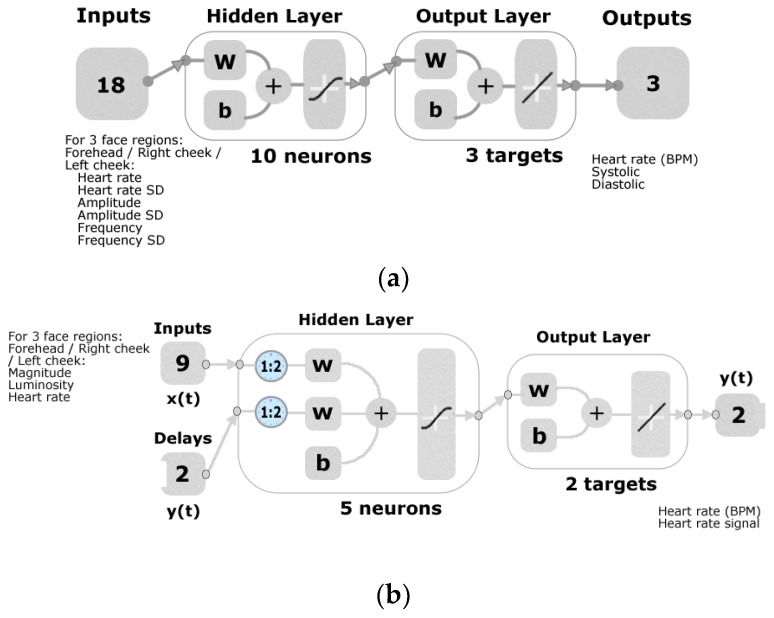
Diagrams of the machine learning models developed where: (**a**) shows Model 1 for regression fitting with a two-layer feedforward network and tan-sigmoid function in the hidden layer and a linear transfer function in the output layer, in which a total of 18 inputs and three targets/outputs used with ten hidden neurons, and (**b**) Model 2 using the shallow neural network time-series prediction with a series-parallel architecture using a tan-sigmoid function in the hidden layer and a linear transfer function in the output layer, nine inputs, two delays, two targets/outputs and five hidden neurons. Abbreviations: SD = standard deviation, w = weights and b = biases.

Data from the video analysis of three face regions (forehead, right and left cheeks) with the RVA were used as inputs along with data from the oscillometric monitor and finger sensor as targets to develop two different machine learning models in Matlab^®^. Model 1 was developed for discriminate analysis of averaged HR by testing 17 different artificial neural network (ANN) training algorithms using an automated customized code written in Matlab^®^. The algorithms tested were two backpropagation using Jacobian derivatives, 11 backpropagation using gradient derivatives, and four supervised weight/bias training (data not shown). The customized code was able to develop a regression model using 18 inputs (six per face region), normalized from −1 to 1, from the video analysis from each of the 17 algorithms tested with inputs such as: (i) average HR in BPM calculated from the video, (ii) standard deviation of the HR, (iii) amplitude of the peaks from the signal of the luminosity changes, (iv) standard deviation of the amplitude form the peaks, (v) average distance or frequency of the peaks from the signal of the luminosity changes, and (vi) standard deviation of the distance or frequency of the peaks, and three targets/outputs from the oscillometric monitor: HR in BPM, systolic (SP) and diastolic blood (DP) pressure. The best model was obtained by using the Levenberg-Marquardt backpropagation training algorithm and random data division by using 70% (*n* = 31) of the samples for training, 15% (*n* = 7) for validation with a mean squared error performance algorithm and 15% (*n* = 7) for testing with a default derivative function. After training the model several times using the neuron trimming technique (3, 5, 7 and 10), it was defined that ten neurons were needed to develop a more accurate model, as shown in Figure 3a. The distribution of the error was tested for normality (*p* ≥ 0.05) through the DAgostino & Pearson Test using the “normalitytest” function in Matlab^®^ R2018a [36].

The PS values, which are the process settings of the normalization (−1–1) of the targets in Matlab^®^ were saved; these values allow to denormalize the new outputs when the model is fed with new inputs. Therefore, the model will give three outputs which will consist of one value for systolic pressure, one for diastolic pressure and one for heart rate, the first two values in mmHg and HR in BPM.

Model 2 for continuous analysis of video data was developed using the shallow neural network time-series prediction (SNNTS) application in the Matlab Neural Network Toolbox 11.1™ (Mathworks Inc., Matick, MA, USA) and the non-linear autoregressive with exogenous input (NARX). The HR, magnitude and luminosity data obtained from the video analysis using the RVA from every second of each video and from each participant for each of the three face regions (forehead, right and left cheeks) were used as normalized (−1 to 1) inputs. The continuous (every second) raw signal of the luminosity changes and HR values obtained using the finger sensor were used as targets/outputs. The use of HR values obtained from the video analysis using the RVA as part of the inputs to predict the HR measured with the finger sensor is to obtain the real HR values and increase the accuracy of the results. The model was developed using the Levenberg-Marquardt backpropagation training algorithm with 70% (*n* = 7688) of the data used for training, 15% (*n* = 1648) for validation with a mean squared error performance algorithm and 15% (*n* = 1648) for testing with a default derivative function; all data were divided using a random data division function. A total of five hidden neurons were selected after retraining the model several times using the neuron trimming technique (3, 5, 7 and 10) as similar results were obtained using five, seven and ten neurons. Furthermore, two delays were used to develop the model (Figure 3b).

Statistical data to evaluate the accuracy of both models was based on the correlation coefficient (R), performance based on the mean squared error (MSE) for each stage (training, validation, testing and overall model), and slope and *p*-value of the overall model calculated using Matlab^®^.

### 2.4. Application of HR and BP to Sensory Analysis of Chocolates

Video and self-reported data from five consumers were extracted from a sensory session with three different chocolate samples with distinct sweeteners (Control: sugar; Treatment 1: D-tagatose; Treatment 2: Stevia). These data were obtained to show a potential application for the presented method. This test was conducted on a different date than the HR validation session and without the use of monitors or sensors attached to the body. Similar to the data obtained for the HR validation test explained in Section 2.1, this session was conducted in the same individual booths presenting a questionnaire in the tablet displaying the bio-sensory App while recording the video from the participant while tasting the samples. The App presented questions to assess acceptability of the samples according to the following descriptors: (i) liking of cocoa aroma (ACocoa), (ii) liking of sweetness (Tsweet), (iii) sweetness intensity with just about right (JAR) test (JARSweet), (iv) liking of bitterness (TBitt), (v) JAR of bitterness intensity, (vi) liking of hardness (Hard), (vii) liking of smoothness (Smooth), (viii) overall liking, and (ix) purchase intention (PurInt). The videos recorded during the session were then analyzed using the RVA method and further processed using Model 1 to obtain the HR and BP values. A correlation matrix was developed using the nine descriptors from the self-reported responses (conscious) along with the HR, and systolic and diastolic pressure (SP and DP, respectively) to assess significant (*p* < 0.05) correlations among the different parameters.

## 3. Results

### 3.1. Matlab^®^ Codes Performance

The algorithm that presented the lowest performance and the highest processing time was the EMA for the three different computers tested, with and without the parallel pool option, being the Alienware with 32 GB and ten cores without parallel pool the fastest (1424 s) and the lowest in CPU usage (14%) (Figure S1a). Comparing the HR analysis from both the EMA and RVA (Figure S1b and S1c), the Alienware with 32 GB and 10 cores with parallel pool and using the RVA presented the best performance with 40 s, 7% of CPU usage and with low maximum temperatures (27–33 °C) in the individual cores. The HP with 8 GB and 2 cores both with and without parallel pool was the lowest in performance for the three codes due to the limited processing capacity of the device. Although both Alienware devices with four and ten cores are within the most powerful gaming computers, the Eulerian magnification algorithm (EMA) still required considerably high processing times of over 1400 s. Furthermore, both Alienware computers had higher memory usage than HP due to the different capacity of the three devices (8 GB vs. 32 GB).

### 3.2. Correlations between Methods

The comparison between the HR obtained by oscillometric monitor and those measured with the finger sensor, both in beats per minute (BPM) rendered a statistically significant and high positive linear correlation, as expected (R^2^ = 0.97; RMSE = 2.65; *p*-value < 0.0001). Hence, both results may be used as a reference of the real HR values for validation purposes.

**Figure 4:**
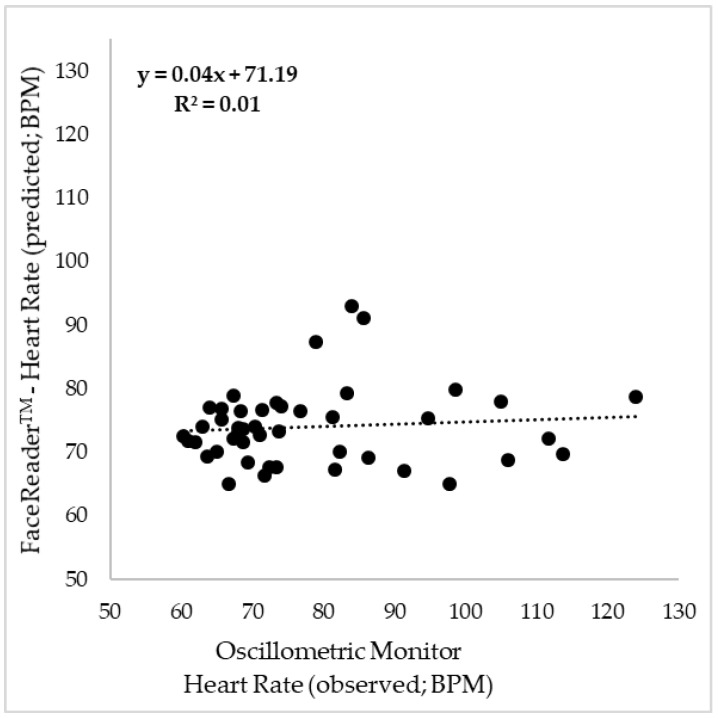
Correlation between the HR measured using the oscillometric monitor (x-axis) and the video analysis using FaceReader™ (y-axis) in beats per minute (BPM). R^2^ = determination coefficient.

Figure 4 shows the results from the means of the HR values from each video of each participant obtained using FaceReader™ (y-axis) and the means from the three measurements obtained using the blood pressure monitor during each video (x-axis). The correlation between both methods was non-significant and with an extremely low determination coefficient (R^2^ = 0.01; RMSE = 230.43; *p*-value = 0.55).

**Figure 5:**
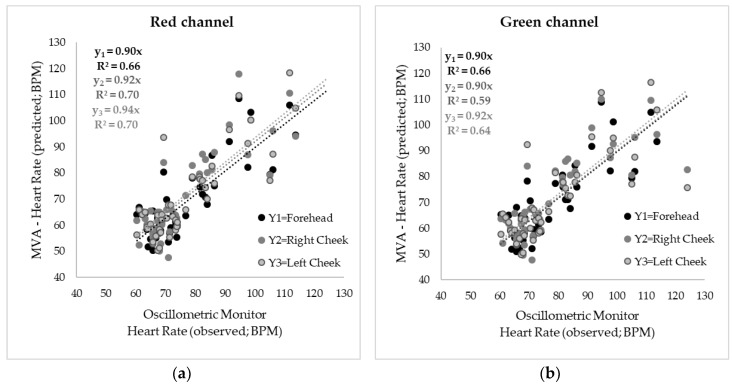

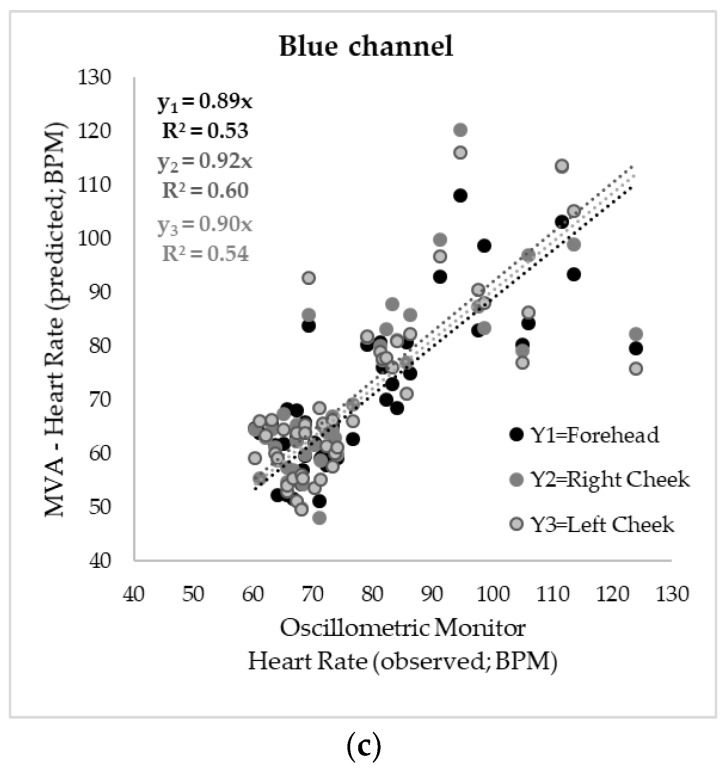
Correlations between heart rate (HR) measured using the oscillometric monitor (x-axis) and video analysis using Matlab^®^ and the pre-processed videos with the Eulerian Magnification algorithms (MVA) (y-axis) in beats per minute (BPM). The figure shows the results of the videos analyzed using the luminosity changes in (**a**) the red channel, (**b**) the green channel and (**c**) the blue channel. R^2^ = determination coefficient.

The means from the results from the videos using the MVA (y-axis) against the mean values from the oscillometric monitor (x-axis) are shown in Figure 5. The videos were analyzed six times as follows: three times to measure the HR in each of the three face regions (forehead, right cheek and left cheek) and three more times by setting the different color channels from the RGB component for each face region. Figure 5a shows the results using the red (R) channel showing a significant correlation (*p*-value < 0.0001) for the three face regions with R^2^ = 0.66 and RMSE = 8.50 for the forehead (y_1_), R^2^ = 0.70 and RMSE = 9.06 for the right cheek (y_2_) and R^2^ = 0.70 and RMSE = 9.07 for left cheek (y_3_). In Figure 5b the correlations using the green (G) channel can be observed with the same determination coefficient as the R channel for the forehead (R^2^ = 0.66; RMSE = 8.44; *p*-value < 0.0001) and lower values for right (R^2^ = 0.59; RMSE = 8.19; *p*-value < 0.0001) and left (R^2^ = 0.64; RMSE = 8.95; *p*-value < 0.0001) cheeks. Figure 5c shows the results from the video analysis using the blue (B) channel where low correlations, yet significant (*p*-value < 0.0001) for the three face regions can be observed with R^2^ = 0.53 and RMSE = 9.56 for the forehead, R^2^ = 0.60 and RMSE = 10.37 for the right cheek, and R^2^ = 0.54 and RMSE = 10.97 for the left cheek.

**Figure 6:**
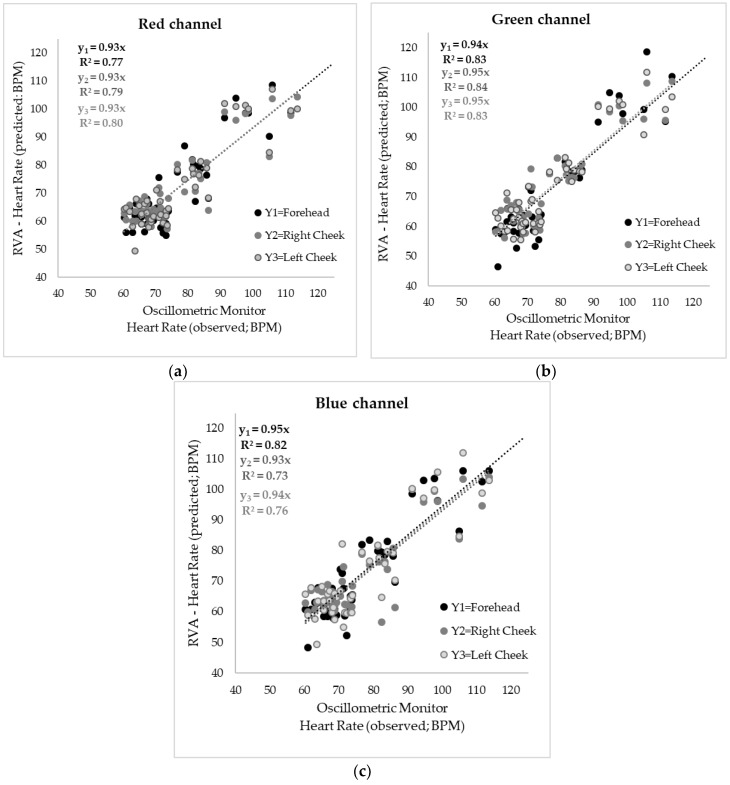
Correlations between heart rate (HR) measured using the oscillometric monitor (x-axis) and video analysis using the raw video HR algorithm (RVA) (y-axis) in beats per minute (BPM). The figure shows the results of the videos analyzed using the luminosity changes in (**a**) the red channel, (**b**) the green channel and (**c**) the blue channel. R^2^ = determination coefficient.

Figure 6 shows the results of the means of the HR measured using the oscillometric monitor (x-axis) and those from the videos analyzed using the proposed RVA method for each video of each participant. All videos were analyzed three times for each of the face areas (forehead, right cheek and left cheek) and using each of the three color-channels in the RBG color spectrum. As shown in Figure 6a, when analyzing the videos for the luminosity changes in the red channel, the three areas had high and significant correlation (*p*-value < 0.001) with R^2^ = 0.77 and RMSE = 7.13 for the forehead results, R^2^ = 0.79 and RMSE = 6.27 for the right cheek values, and R^2^ = 0.80 and RMSE = 6.74 for the left cheek. In Figure 6b the results using the green channel can be observed, where the forehead results presented a significant correlation with a high determination coefficient R^2^ = 0.83 (RMSE = 7.26; *p*-value ≤ 0.0001), likewise the right and left cheeks showed a high and significant correlation (*p*-value < 0.0001) with R^2^ = 0.84, RMSE = 6.05 and R^2^ = 0.83, RMSE = 6.37, respectively. The videos analyzed using the luminosity changes in the blue channel had a lower, yet significant (*p*-value < 0.0001) correlation for the three face areas (Figure 6c). Using blue channel, the forehead showed the highest determination coefficient from the three areas (R^2^ = 0.82, RMSE = 6.72), followed by the left cheek (R^2^ = 0.76, RMSE = 7.61) and the right cheek (R^2^ = 0.73, RMSE = 7.12).

### 3.3. Machine Learning Modelling

**Figure 7:**
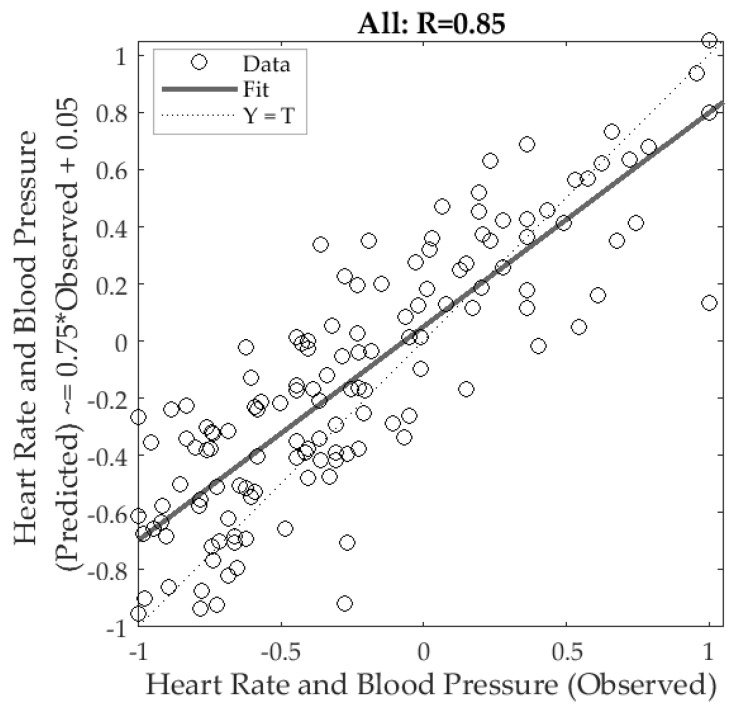
The artificial neural network regression overall model (Model 1) showing a high correlation coefficient (R = 0.85), where the x-axis represents the observed (targets) and the y-axis represents the predicted (outputs) values of heart rate (HR) and blood pressure (BP) measured with the oscillometric monitor. All values were normalized in a scale from −1 to 1.

Figure 7 shows the Model 1 developed using the artificial neural networks regression modelling. It can be observed that is was possible to develop a significant (*p*-value < 0.0001) overall model with high correlation (R = 0.85) and a slope of 0.75 using the 18 inputs obtained from the video analysis with the RVA presented in this paper to predict the HR and BP values together measured using the oscillometric monitor. The number of samples, performance based on the mean squared error (MSE) and correlation coefficients for each stage are shown in Table 1. Based on these results, the testing stage had the lowest correlation (R = 0.71), the three stages had a performance MSE of 0.07 for training, 0.13 for validation and 0.11 for testing stages at epoch two. Both the validation and testing performance are similar and presented a decrease trend, which indicates that there was no overfitting in the model. The errors of the three stages (training, validation and testing) presented a normal distribution (*p* ≥ 0.05; D’Agostino & Pearson Test = 0.07).

**Figure 8:**
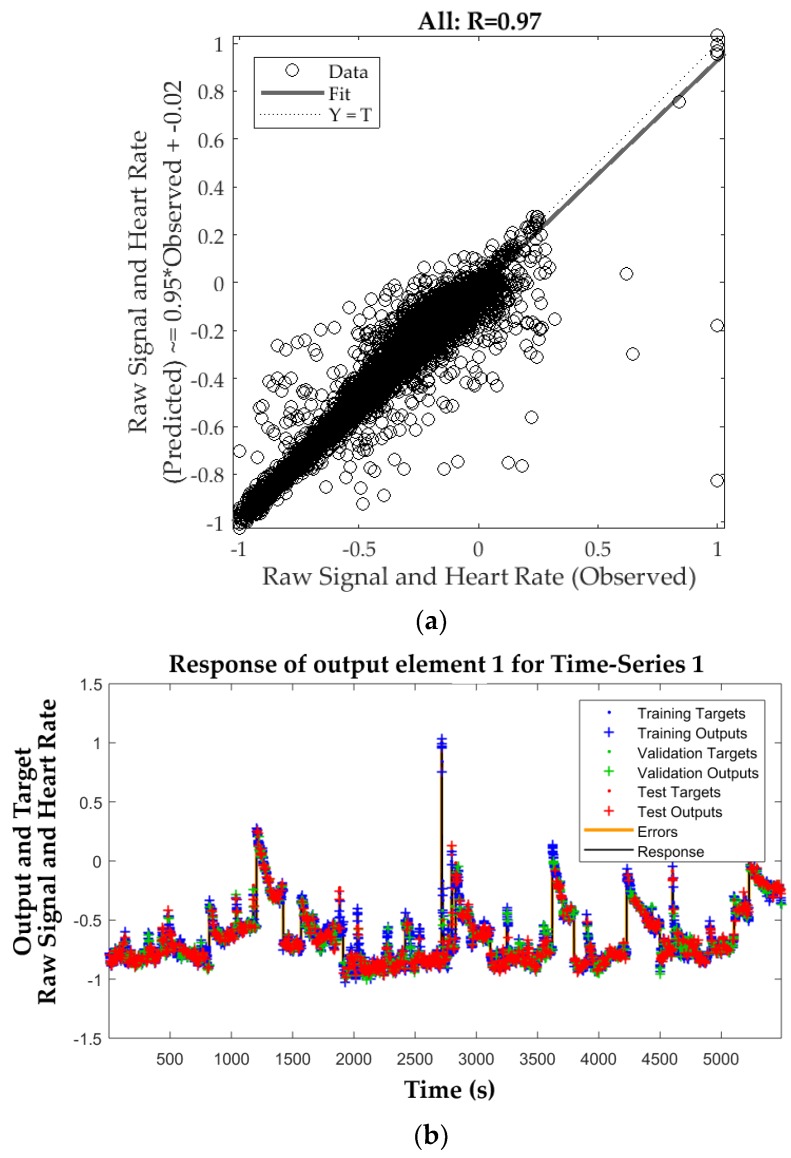
Response from the shallow neural network time-series (SNNTS) model (Model 2) where: (**a**) shows the regression model with an overall high correlation (R = 0.97), where the x-axis represents the targets (observed) and the y-axis depicts the outputs (predicted) values of the raw signal of the luminosity changes and HR measured using the finger sensor. All values were normalized in a scale from −1 to 1; (**b**) shows the graph with the response output for the time series for every 1 second comparing the targets and outputs where x-axis represents the time (s) and the y-axis the normalized (−1 to 1) response values of raw signal of the luminosity changes and real HR (HR).

The SNNTS model (Model 2) is shown in Figure 8. This model used nine inputs from the video analysis using the RVA to predict the real HR and raw signal values of the luminosity changes every one second. It can be observed that the overall model presented a significant (*p*-value < 0.0001) and high correlation coefficient (R = 0.97) with a slope of 0.95 (Figure 8a). Figure 8b shows the response of outputs every 1 second for both the target (observed) and output (predicted) values. Table 2 shows the mean squared error performance values and correlation coefficient for each stage and the overall model. All three stages and overall model presented similar correlation coefficients and had a performance of MSE = 0.004 at epoch 44. Both the validation and testing performance did not increase before the iteration 44, which is an indicator of no overfitting in the model. The correlations of the errors as well as those from the input-error cross-correlation fell within the 95% confidence limits, which validate the network performance (data not shown).

### 3.4. Application of HR and BP Responses (Model 1) to Sensory Analysis (Chocolates)

**Figure 9:**
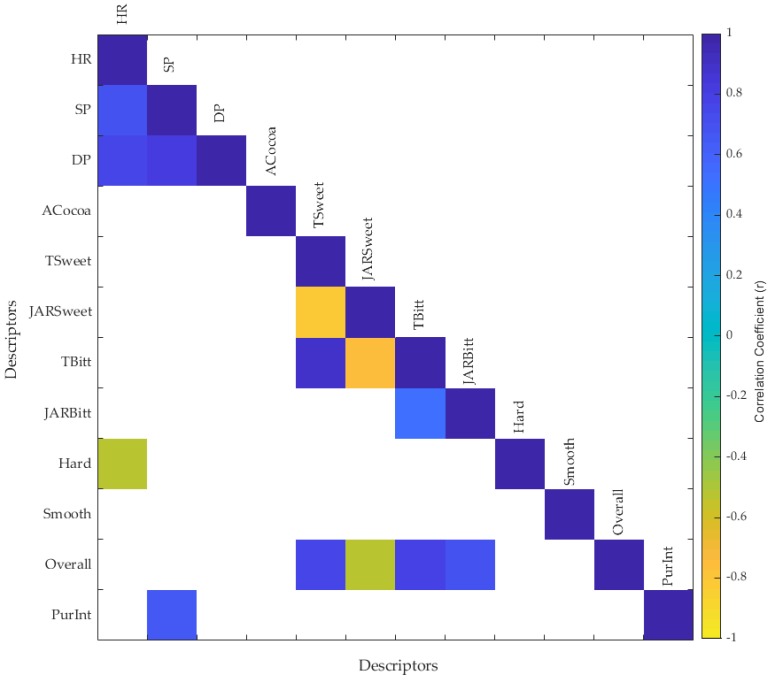
Correlation matrix of a chocolate sensory test showing the significant correlations (*p* < 0.05) in which the blue side of the color scale (−1 to 1) represent the positive and the yellow side the negative correlations. Abbreviations: (i) heart rate (HR), (ii) systolic pressure (SD), (iii) diastolic pressure (DP), (iv) liking of cocoa aroma (ACocoa), (v) liking of sweetness (Tsweet), (vi) sweetness intensity with just about right (JAR) test (JARSweet), (vii) liking of bitterness (TBitt), (viii) JAR of bitterness intensity, (ix) liking of hardness (Hard), (x) liking of smoothness (Smooth), (xi) overall liking, and (xi) purchase intention (PurInt).

Figure 9 shows the correlation matrix of the sensory session with the chocolate samples. The HR had a positive and significant correlation with SP and DP (R = 0.67; R = 0.74) and a negative correlation with the liking of hardness of the chocolate (R = −0.54). On the other hand, the SP had a positive correlation with DP (R = 0.80) and importantly with purchase intention (PurInt; R = 0.65).

## 4. Discussion

### 4.1. Matlab^®^ Codes Performance

From the results of the computer and Matlab^®^ performance to calculate HR using videos, it can be observed that the MVA method presented the lowest performance as it required above 2300 s of processing time, for both algorithms (EMA + MVA), to process a single video, and have a high memory (~800 MB) and CPU usage (~35%) when running them in a computer with 8 GB and 2 cores. The performance for this method improves with the use of more powerful computers, however, the processing times and memory usage are still high (>1500 s; ~1800 MB), especially if the user needs to analyze several videos, this would be the case for a sensory session in which usually a minimum of 40 participants and around four to six samples (>160 videos) are required. Therefore, the video analysis using the RVA is less-time consuming and present a more efficient method to calculate HR as it can be processed using a computer with less capacity.

### 4.2. Correlations between Methods and Real HR

Even though the finger sensor and the oscillometric monitor use a different method to measure HR, photoplethysmography (PPG) versus oscillometry, both methods are usually used for medical purposes and presented a high and significant correlation when comparing both (R^2^ = 0.97), which shows that both are appropriated to be used for validation purposes. In this paper both methods were used, as the PPG is able to measure the HR and record the raw signal of the luminosity changes continuously, while the oscillometric monitor is able to measure HR and blood pressure, but only gives one value for every measurement.

When comparing the HR results from the oscillometric monitor with those obtained by analyzing the videos through FaceReader™ there was no correlation between the two methods (R^2^ = 0.01; *p*-value = 0.55). This contradicts the study from Tasli et al. [23] in which the authors mention that there is a high correlation between the HR measured with a pulse oximeter attached to the finger and the FaceReader™ HR method, however, they did not report the statistical data such as R^2^ and *p*-value. Another weakness found in that study was that the authors used only ten participants and recorded two videos of each (20 videos total) which is a small sample size compared with the present study, in which 15 participants and three videos of each (45 videos total) were used. From the Figure 3 it can be seen that the major fail of the FaceReader™ method is in the high HR values as it underestimates the results, this was also shown in the study by Tasli et al. [23] as in the results they presented, there is a larger number of points outside the confidence bounds in the high HR range.

Another method using video analysis tested in this study was the MVA, which involved the pre-processing of the videos using the Eulerian Magnification algorithm, the correlations using the three color-channels (RGB) and the three face regions resulted in low to moderately high (R^2^ = 0.53 to 0.70), being the red channel the highest in accuracy. However, compared to the analysis of the raw videos with the RVA, the correlations using the MVA were much lower. The best correlations using the RVA were with the green channel (R^2^ = 0.83) and this was similar for the three face regions. Studies from other authors such as Carvalho et al. [16] who proposed a method for video analysis using the EMA showed that the values obtained are close to those measured using a HR monitor, nevertheless, it was only tested with five participants (one value of each), therefore, their sample size was too small to assess the accuracy of their method. Wu et al. [17] developed the Eulerian Magnification method and tested it obtaining similar values to medical HR devices, but they measured it using only three videos (from two adults and one baby) which is not enough to validate a method and did not present any values and statistical analysis. On the other hand, authors such as Jensen and Hannemose [18] showed the comparison of different methods to calculate HR using the raw videos and different color models and showed a high correlation R > 0.95 using the green channel and 30 s videos at 15 frames per second (fps) from 12 participants. These authors also showed that the highest blood flow can be found in the cheeks and forehead. The green channel works best due to the capacity oxy- and deoxyhemoglobin to absorb the green light, which allows to measure the blood flow in the skin surface, this method is also used by the finger sensor tested in this study.

### 4.3. Machine Learning Modelling

In this study, an artificial neural network (ANN) regression model (Model 1) was used to estimate the real HR and BP values using the oscillometric monitor results as targets, and the average of the results (HR, frequency and amplitude) and their standard deviation from each face region obtained from the RVA as inputs. The high correlation (R = 0.85) obtained in the overall model shows that it is feasible to predict HR and blood pressure (systolic and diastolic). This model can be used to obtain the average of these physiological responses in consumer sensory tests when evaluating food, beverages and/or packaging to assess differences in responses to distinct samples. Authors such as Jain et al. [15] have developed a method to estimate HR and BP using computer vision based on the extraction of the PPG signal, principal components analysis, peak detection algorithms and polynomial kernel regression modelling, and compared their method with a blood pressure monitor similar to the one used in the present study, obtaining high accuracy. However, the ANN models are able to learn and detect more patterns within data when more samples are added to the inputs, which enables the model to improve its performance. Furthermore, ANN has been successfully used in different fields such as to predict cardiac diseases based on HR data [37,38], to classify level of acceptability of beer using non-intrusive biometrics (HR, body temperature, facial expressions and brainwaves) [5], among others.

A different machine learning method was used to develop Model 2, in which a shallow neural network time-series technique was used to predict the real PPG raw signal of the luminosity changes and HR values every second using some of the results obtained from the RVA (HR, luminosity and magnitude) from each face region as inputs. This model, which resulted highly accurate (R = 0.97), would be useful for sensory studies involving the assessment of physiological responses of consumers to stimuli for some specific tests for food tasting such as temporal dominance of sensations (TDS), time intensity sensory tests or while using videos as stimuli in which the responses (conscious and unconscious) need to be measured over time.

### 4.4. Application of HR and BP Responses to Sensory Analysis (Chocolates)

Results from the sensory analysis of chocolate for consumers’ acceptability showed that it is possible to find a relationship between the self-reported (conscious) and the physiological responses as there was a decrease in HR when consumers disliked the hardness of the chocolate samples. On the other hand, an increase in the SP was detected when consumers are more willing to purchase the product. Therefore, these findings can be used as an example of a potential application of the technique presented in this paper, however, besides food products, it may also be used along with other biometrics such as body temperature and facial expressions to obtain more information from consumers for sensory analysis of beverages [5,6] and packaging. Up to date, there are no studies including BP as a physiological response to food or beverages, therefore, the method presented in this paper, proposes BP as an additional parameter to explore deeper for this application. Therefore, further validations must be performed to assess the variability or changes in BP when evaluating different food and beverages.

## 5. Conclusions

The proposed method using RVA which works by analyzing raw videos using the luminosity changes in the green channel along with machine learning algorithms based on artificial neural network regression to predict HR and blood pressure and shallow neural networks time-series prediction to estimate HR and raw signal from luminosity changes every second showed to be an accurate and reliable technique to measure physiological responses of subjects. This non-contact method can be used as a biometric tool to assess consumers’ responses when evaluating different stimuli such as food, beverages and packaging to complement other unconscious and conscious responses that would allow to obtain more information in sensory analysis. This technique also has the advantage that it presented the best performance when using computers with different memory and number of cores and requires less processing times than other methods such as the pre-processing of videos using Eulerian magnification algorithms. Further studies will consist on the implementation of an automatic algorithm to crop and track the specific face regions that would allow to optimize the method and improve the accuracy of the results and models.

## Figures and Tables

**Table 1:** Statistical results of Model 1 showing the number of samples, mean squared error (MSE) and correlation coefficient (R) for the training, validation and testing stages as well as the overall model.

Stage	Samples	Mean Squared Error (MSE)	Correlation Coefficient (R)
Training	31	0.07	0.89
Validation	7	0.13	0.72
Testing	7	0.11	0.71
Overall	45	0.06	**0.85**

**Table 2:** Statistical results of Model 2 showing the number of samples, mean squared error (MSE) and correlation coefficient (R) for the training, validation and testing stages as well as the overall model.

Stage	Samples	Mean Squared Error (MSE)	Correlation Coefficient (R)
Training	7688	0.004	0.97
Validation	1648	0.004	0.98
Testing	1648	0.004	0.98
Overall	10,984	0.004	**0.97**

## Supplementary Materials

The following are available online at http://www.mdpi.com/1424-8220/18/6/1802/s1, Figure S1: Comparison of the performance of different computers tested using the Matlab® codes to (**a**) pre-process the videos magnifying the color changes with the Eulerian Magnification Algorithm (EMA), and to calculate the HR values using: (**b**) the Magnified Videos (MVA) and (**c**) the Raw Videos (RVA). Results using different computer brands and with distinct specifications are presented. The values shown are normalized from 0 to 1 and shown in a color scale from white to black in which the black corresponds to the highest values and white the lowest. The duration of the video used to obtain the performance results was 02:51 min. Abbreviations: HP = Hewlett Packard, AW = Alienware, P = parallel pool, NP = no parallel pool, MaxT = maximum temperature and MinT = minimum temperature.
