# Supplementary material for: Non-Contact Heart Rate and Blood Pressure Estimations from Video Analysis and Machine Learning Modelling Applied to Food Sensory Responses: A Case Study for Chocolate
Source: Sensors (Basel). 2018 Jun 3;18(6):1802. doi: 10.3390/s18061802 (PMC6022164; doi:10.3390/s18061802)
Supplement: Supplementary file 1 [file sensors-18-01802-s001.pdf]

Sensors **2018**, *18*, 1802; doi:10.3390/s18061802 www.mdpi.com/journal/sensors

Non-Contact Heart Rate and Blood Pressure Estimations from Video Analysis and Machine Learning Modelling Applied to Food Sensory Responses: A Case Study for Chocolate

Claudia Gonzalez Viejo, Sigfredo Fuentes *, Damir D. Torrico and Frank R. Dunshea

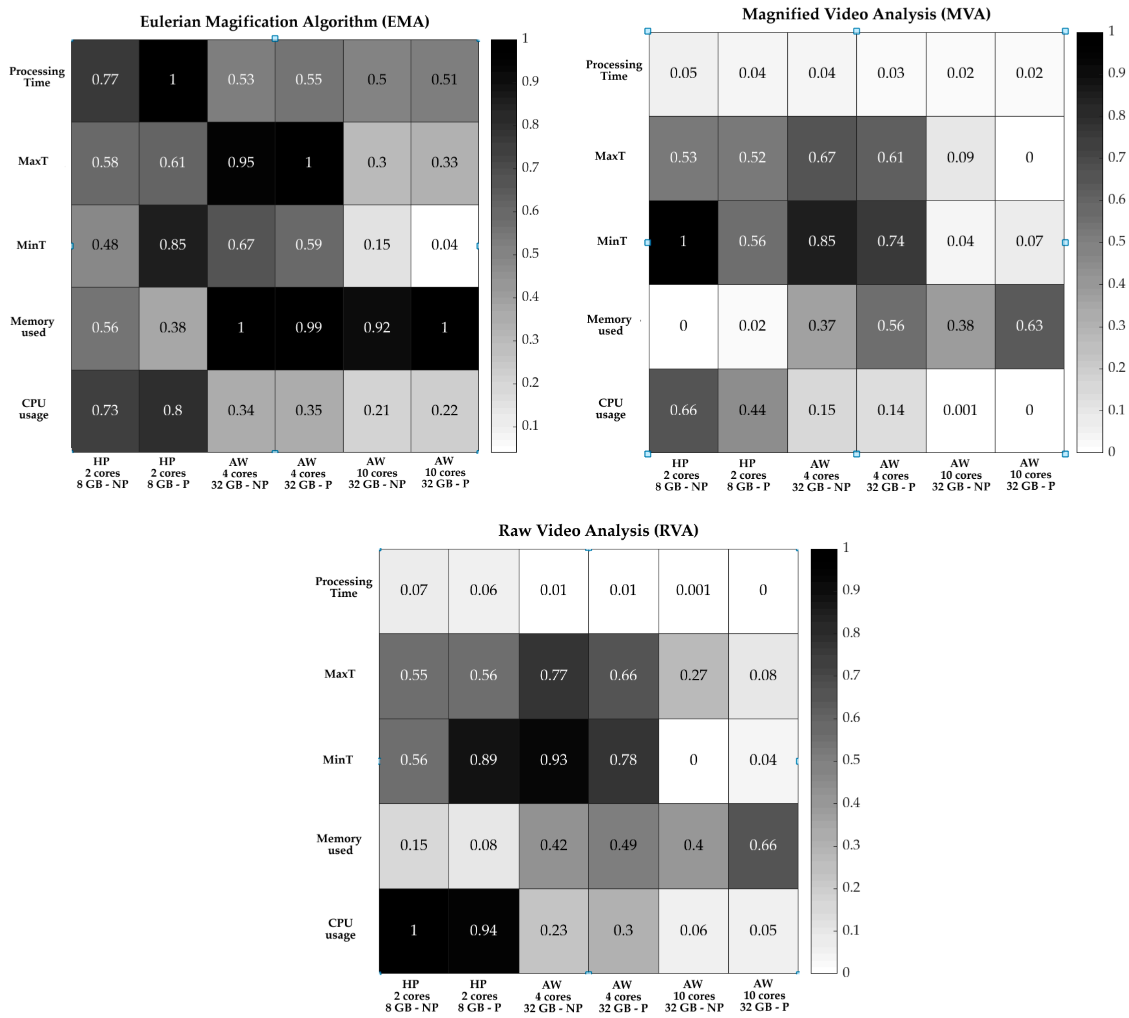

Figure S1. Comparison of the performance of different computers tested using the Matlab® codes to (a) pre-process the videos magnifying the color changes with the Eulerian magnification algorithm (EMA), and to calculate the HR values using: (b) the magnified videos (MVA) and (c) the raw videos (RVA). Results using different computer brands and with distinct specifications are presented. The values shown are normalized from 0 to 1 and shown in a color scale from white to black in which the black corresponds to the highest values and white the lowest. The duration of the video used to obtain the performance results was 02:51 min. Abbreviations: HP = Hewlett Packard, AW = Alienware, P = parallel pool, NP = no parallel pool, MaxT = maximum temperature and MinT = minimum temperature.
